# CustFRE: An annotated dataset for extraction of family relations from English text

**DOI:** 10.1016/j.dib.2022.107980

**Published:** 2022-02-19

**Authors:** Raabia Mumtaz, Muhammad Abdul Qadir, Asif Saeed

**Affiliations:** a Capital University of Science & Technology, Islamabad, Pakistan; b COMSATS University Islamabad, Attock Campus, Pakistan

**Keywords:** Natural language processing, Relation classification, Machine learning, Family relations

## Abstract

Meaningful Information extraction is an extremely important and challenging task due to the ever growing size of data. Training and evaluating automated systems for the task requires annotated datasets which are rarely available because of the great amount of human effort and time required for annotating data. The dataset described in this manuscript, CustFRE, is meant for systems that learn extracting family relations from text. Sentences having at least two persons have been collected from the internet. The texts are first processed using Stanford's NLP pipeline for basic NLP tagging. Next, a team of natural language processing experts annotated the dataset. All family relations among persons in the texts have been annotated, or a no_relation is annotated if no family relation between two persons can be inferred from the text. After annotation, the dataset was verified by an NLP expert for completeness and correctness. CustFRE contains in total 2,716 annotations. The dataset can be used by information extraction researchers as a benchmark for evaluating their systems, and can also be used for training and evaluating family relation extraction systems.

## Specifications Table

**Table:** 

Subject	Computer Science
Specific subject area	Information extraction
Type of data	Table
How the data were acquired	Sentences for annotation were collected from the internet, then processed using Stanford NLP pipeline, and manually annotated with family relation tags.
Data format	Analysed
Description of data collection	Only those sentences were collected for annotation, which have at least two persons, as family relations exist between persons. Collected data was further processed using Stanford NLP pipeline to add part of speech, named entity recognition and dependency relation tags. Family relation labels for six classes (per:children, per:parents, per:spouse, per:siblings, per:other_family, no_relation) were manually added.
Data source location	Sentences for annotation are collected randomly from the following public websites: https://americanliterature.com/100-great-short-stories https://dunyanews.tv/ https://www.bbc.com/news https://www.theguardian.com/ https://www.timesnews.net/ http://www.wikipedia.org
Data accessibility	Repository name: Mendeley Data Data identification number: 10.17632/jps7rfkytr.1 Direct URL to data: https://data.mendeley.com/datasets/jps7rfkytr/1

## Value of the Data

• This dataset is useful for building automated systems, whether machine learning based or rule based, for extracting family relations from English text.
• Knowledge and Information extraction researchers can benefit from this dataset in developing systems for Knowledge base population.
• The dataset can be considered as a benchmark for evaluating systems developed for family relations classification.
• To the best of our knowledge, no such comprehensive dataset exists in public domain to facilitate research work in this field.

## 1 Data Description

**Fig. 1:**
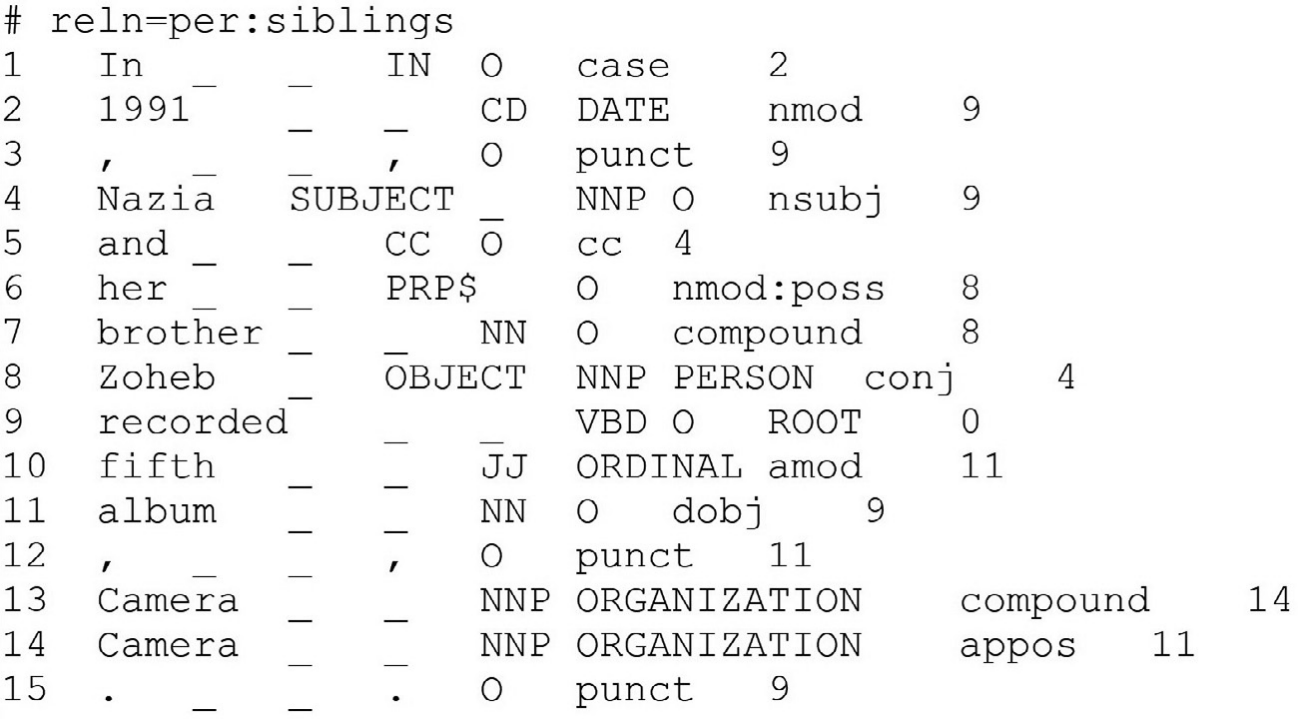
An excerpt from the dataset conll file.

The presented dataset is prepared for the purpose of building and evaluating various methods for the task of extracting and classifying family relations from input sentence. Formally, a family relation is a subject, predicate, object triple where the predicate is a family relation. The dataset in total contains 2,716 annotations and is provided in two formats, conll and json. Fig. 1 presents an example excerpt from the dataset in conll format. Each example's first line is # reln=family-relation, where family-relation is the family relation out of the six classes annotated for this example. Each next line represents a token of the example sentence and contains eight tab separated attributes; the index of token, the token, SUBJECT if the token is subject – otherwise, OBJECT if the token is object – otherwise, the Stanford's part of speech tag of the token, the Stanford's named entity recognition tag of the token, the Stanford's dependency relation tag of the token, and the Stanford's dependency head of the token. Examples in conll file are separated by blank lines.

**Fig. 2:**
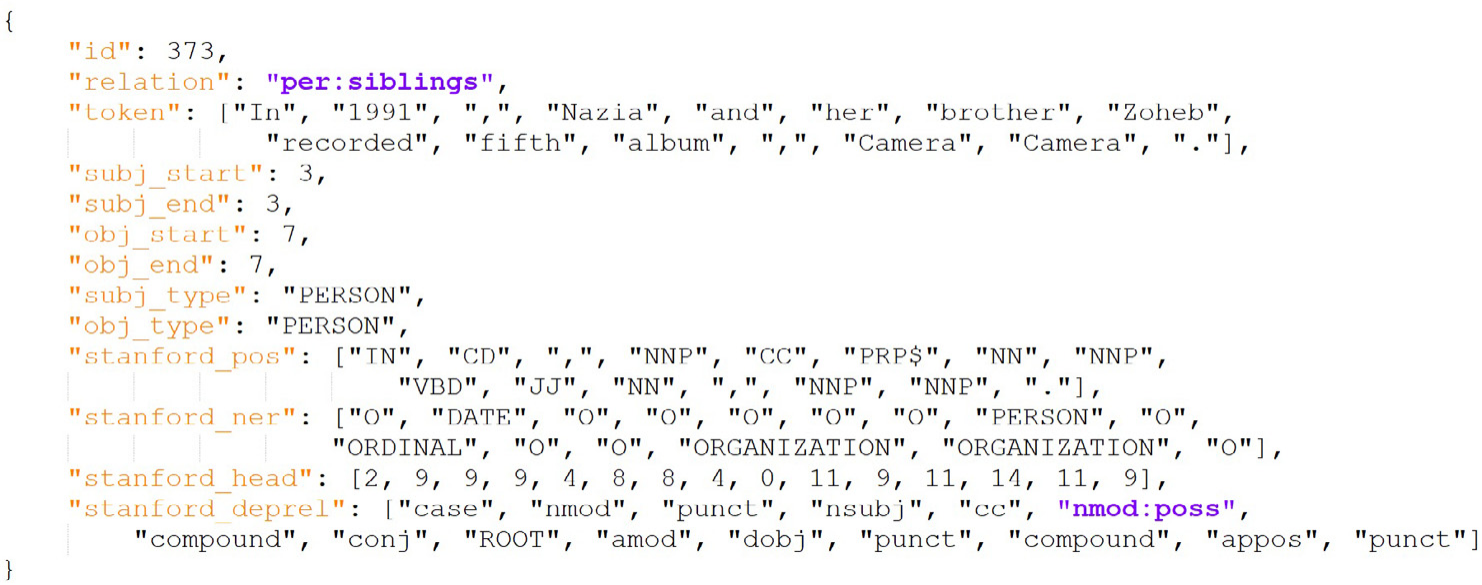
An excerpt from the dataset json file.

Fig. 2 presents an example excerpt from the dataset in json format. The file contains an array of examples. Each example contains these twelve attributes, an id which is the example number of this example, the family relation out of the six classes annotated for this example, the tokens of this example, subject start index, subject end index, object start index, object end index, subject type, object type, the Stanford's part of speech tags of this example, the Stanford's named entity recognition tags of this example, the Stanford's dependency relation tags of this example, and the Stanford's dependency heads of this example.

Along with the dataset files, two supplementary files are also provided for convenience: An excel file listing the individual sentences used in the dataset, alongwith the number of persons/relations in each of these (248) sentences, and a relations key text file listing the desired output family relations, one on each line.

A total of 2,716 annotations have been made in the dataset. Average sentence length of CustFRE, in terms of number of tokens, is 35. Number of examples of each type of relation in the dataset are given in Table 1.

**Table 1:** Relation Distribution of the CustFRE Dataset.

	Family Relation Class						
	children	other_family	parents	siblings	spouse	no_relation	All
**No of examples**	347	302	347	282	263	1,175	2,716
**Percentage of total**	13%	11%	13%	10%	10%	43%	100%

## 2 Experimental Design, Materials and Methods

The sentences for the dataset have been collected from three type of online sources, short stories (https://americanliterature.com/100-great-short-stories), Wikipedia articles (https://en.wikipedia.org/wiki/) and from online news and magazines websites (https://dunyanews.tv/, https://www.bbc.com/news, https://www.theguardian.com/, https://www.timesnews.net/). Collecting examples of family relations was a difficult task as these examples are not generally found in usual texts. Examples of family relations can be found in such texts as stories about persons, biographies of persons, news about persons, etc. Therefore, for collecting relevant examples, online stories, Wikipedia articles about persons, and family news articles from news websites were manually skimmed and relevant sentences were collected. A total of 248 sentences have been collected and each sentence contains two or more persons.

The collected sentences are first processed using Stanford's NLP pipeline [1] and tagged for parts of speech, named entity recognition, and dependency parsing. Output from Stanford is taken in conll format. Next, a team of natural language processing (NLP) researchers annotated these sentences for family relations. The pre-defined relation vocabulary used for annotation contains the family relations used in the well-known TAC KBP challenges [2] and in TACRED [3]. These are, the five family relations, per:spouse, per:children, per:parents, per:siblings, and per:other_family, and no_relation if none of the five family relations exists between two persons.

We have made sure that in our dataset, all possible relations between persons in a text are annotated. If a text contains ***n*** persons, the number of possible relations between these persons is

$$P\left(n,r\right)=\frac{n!}{\left(n-r\right)!}$$

Where r=2, as we are only concerned with binary relations, i.e. relations between two persons. We count permutations because the direction of relation is important, i.e. the relation of X to Y might be different from the relation of Y to X. A team of NLP researchers annotated each sentence with the P(n,2) possible family relations between the n persons (or person pronouns) of the sentence. For example, the sentence "In 1991, Nazia and her brother Zoheb recorded fifth album, Camera Camera." has three person occurrences (Nazia, her and Zoheb). So for this sentence, the following six relations are annotated in the dataset:

(Nazia, no_relation, her)
(Nazia, per:siblings, Zoheb)
(her, no_relation, Nazia)
(her, per:siblings, Zoheb)
(Zohaib, per:siblings, Nazia)
(Zohaib, per:siblings, her)

After data annotation was completed, an expert, working in the fields of information extraction and NLP for over 30 years, verified the dataset (manually going through every single of 2,716 annotations) and checked that the dataset is correctly and completely annotated and does not contain any erronuous annotations.

## Ethics Statements

The work did not involve any human subject or animal experiments.

## CRediT Author Statement

**Raabia Mumtaz:** Writing- Original Draft, Conceptualization, Methodology, Project Administration, Data curation; **Muhammad Abdul Qadir:** Conceptualization, Methodology, Validation, Supervision; **Asif Saeed:** Data curation.

## Declaration of Competing Interest

The authors declare that they have no known competing financial interests or personal relationships that could have appeared to influence the work reported in this paper.

## Data Availability

CustFRE: An Annotated Dataset for Extraction of Family Relations from English Text (Original data) (Mendeley Data).

## Appendix Supplementary materials

Image, application 1

Image, application 2

Image, application 3

Image, application 4
